# Bioinformatic and molecular investigation of Sirt3 expression

**DOI:** 10.1186/1753-6561-6-S3-P40

**Published:** 2012-06-01

**Authors:** F Kyle Satterstrom, William R Swindell, Martha L Bulyk, Marcia C Haigis

**Affiliations:** 1 Harvard School of Engineering and Applied Sciences, Cambridge, MA 02138, USA; 2 Department of Cell Biology, Harvard Medical School, Boston, MA 02115, USA; 3 The Paul F. Glenn Labs for the Biological Mechanisms of Aging, Harvard Medical School, Boston, MA 02115, USA; 4 Department of Genetics, Harvard Medical School, Boston, MA 02115, USA; 5 Division of Genetics, Department of Medicine, Brigham and Women’s Hospital and Harvard Medical School, Boston, MA 02115, USA; 6 Department of Pathology, Brigham and Women’s Hospital and Harvard Medical School, Boston, MA 02115, USA; 7 Division of Health Sciences and Technology, Harvard Medical School, Boston, MA 02115, USA

## Background

Fasting and calorie restriction cause significant metabolic changes as organisms try to maintain energy homeostasis. The mitochondrial NAD^+^-dependent protein deacetylase Sirt3 has important metabolic effects, including promotion of fatty acid oxidation during fasting [1] and repression of glycolysis in cancer cells [2]. We sought to investigate the mechanisms by which Sirt3 is transcriptionally induced and regulated using both bioinformatic and molecular methods.

## Materials and methods

Our approach was two-pronged: using the DNA sequence analysis program PhylCRM [3], we analyzed the regulatory sequences of Sirt3 and genes with similar expression profiles to determine over-represented transcription factor binding sequences. We also conducted a quantitative real-time PCR-based targeted screen in HEK 293T cells to determine the effects of calorie restriction mimetic drugs on Sirt3 expression levels.

## Results

We have identified candidate transcription factors that may affect Sirt3 expression levels, including the zinc finger transcription factor MZF1. We have also analyzed the effect of several drugs on Sirt3 expression, notably observing a decrease in Sirt3 expression with resveratrol treatment.

## Conclusions

We have identified transcription factors and calorie restriction mimetic drugs which may control Sirt3 expression and are currently conducting follow-up studies.
